# Chronic social stress disrupts the intracellular redistribution of brain hexokinase 3 induced by shifts in peripheral glucose levels

**DOI:** 10.1007/s00109-022-02235-x

**Published:** 2022-08-09

**Authors:** Michael A. van der Kooij, Liliana Rojas-Charry, Maryam Givehchi, Christina Wolf, Diones Bueno, Sabine Arndt, Stefan Tenzer, Lorenzo Mattioni, Giulia Treccani, Annika Hasch, Michael J. Schmeisser, Caterina Vianello, Marta Giacomello, Axel Methner

**Affiliations:** 1 Leibniz Institute for Resilience Research (LIR), Mainz, 55122 Germany; 2 Institute for Molecular Medicine, Johannes Gutenberg University Mainz, Mainz, 55131 Germany; 3 Institute of Anatomy, Johannes Gutenberg University Mainz, Mainz, 55131 Germany; 4 Institute for Immunology, Johannes Gutenberg University Mainz, Mainz, 55131 Germany; 5 Department of Psychiatry and Psychotherapy, Translational Psychiatry, University Medical Center, Johannes Gutenberg University Mainz, Mainz, 55131 Germany; 6 Department of Biology, University of Padua, Padua, 35121 Italy

**Keywords:** Brain, Hexokinase, Mice, Mitochondria, Stress

## Abstract

**Abstract:**

Chronic stress has the potential to impair health and may increase the vulnerability for psychiatric disorders. Emerging evidence suggests that specific neurometabolic dysfunctions play a role herein. In mice, chronic social defeat (CSD) stress reduces cerebral glucose uptake despite hyperglycemia. We hypothesized that this metabolic decoupling would be reflected by changes in contact sites between mitochondria and the endoplasmic reticulum, important intracellular nutrient sensors, and signaling hubs. We thus analyzed the proteome of their biochemical counterparts, mitochondria-associated membranes (MAMs) from whole brain tissue obtained from CSD and control mice. This revealed a lack of the glucose-metabolizing enzyme hexokinase 3 (HK3) in MAMs from CSD mice. In controls, HK3 protein abundance in MAMs and also in striatal synaptosomes correlated positively with peripheral blood glucose levels, but this connection was lost in CSD. We conclude that the ability of HK3 to traffic to sites of need, such as MAMs or synapses, is abolished upon CSD and surmise that this contributes to a cellular dysfunction instigated by chronic stress.

****Key messages**:**

- Chronic social defeat (CSD) alters brain glucose metabolism
- CSD depletes hexokinase 3 (HK3) from mitochondria-associated membranes (MAMs)
- CSD results in loss of positive correlation between blood glucose and HK3 in MAMs and synaptosomes

## Introduction

Long-term stress has the potential to lastingly impair health and may increase the vulnerability for psychiatric disorders such as major depression [1]. Stress readily shapes metabolic processes [2], and a recent line of thinking links alterations in energy metabolism to such effects of chronic stress on the brain [3–5]. Stress hormones can create a chronic state of “metabolic oversupply” involving dysglycemia [6], with the brain representing a particularly vulnerable target [7]. The elevated levels of circulating glucose and lipids during this kind of metabolic stress are known to affect brain structures as well as cognitive function through still ill-defined mechanisms [8, 9] including mitochondrial dysfunction [3, 6, 10].

Chronic stress in animal models recapitulates several functional impairments, reminiscent of depression including cognitive impairments, increased anxiety-like behavior, and reduced reward motivation [11–13]. Chronic social defeat (CSD) is a frequently used social stressor in which a resident mouse defends its territory and attacks intruders. Typically, intruder mice are attacked during a 5–10-min timespan with the resident. To limit morbidity and mortality and enhance reproducibility, we opted for a CSD paradigm which restricts the aggressive duration to 30 s per day for 10 consecutive days. This CSD paradigm results in long-term peripheral and central hyperglycemia [14]. In CSD, peripheral hyperglycemia is coupled to cognitive impairments, suggesting a link between peripheral glucose metabolism and neurofunctional outcome [14]. In stressed animals, hyperglycemia is found in the peripheral blood and in the brain, where it was determined by induced bioluminescence imaging. However, cerebral glucose uptake – measured with the radioactive ligand ^18^fluorodeoxyglucose using positron emission tomography – was reduced. A possible effector of this reduced uptake is the glucose transporter (GluT) 1, which was reduced in the hippocampal synaptic fraction from CSD-exposed mice [14]. Hence, despite an abundance of glucose, the stressed brain may display intracellular alterations reminiscent of glucose hypometabolism or even glucose deprivation. While these potential intracellular adaptations could be interpreted as defense mechanisms, protecting against a stress-induced glucose overload, they may simultaneously be harmful, resulting in neuronal dysfunction.

Mitochondria and the endoplasmic reticulum (ER) are important organelles and nutrient sensors, and their dysfunction has been extensively and independently implicated in metabolic diseases. Both organelles interact at sites known as mitochondria-ER contact sites (MERCs) where they exchange metabolites and calcium [15]. It is therefore feasible that a neuronal dysfunction downstream of metabolic stress might originate at or involve MERCs. The structure and function of MERCs has been intensively studied, but much remains unclear. Some proteins have been identified as structural tethers of MERCs: the IP3R-GRP75-VDAC1 complex, PACS2, and mitofusin 2 (MFN2) oligo/heterooligomers among others [16–18]. Notably, MFN2, which appears to be reduced in response to chronic mild stress or corticosterone exposure [19], has been described as a fundamental mediator of mitochondria-ER communication. An intuitive consequence of this finding would be that these conditions (i.e., chronic stress or/and corticosterone exposure) induce a remodeling of the sites at which mitochondria and ER meet. Importantly, these sites can be isolated through a differential centrifugation procedure. The biochemically isolated fraction of contact sites between mitochondria and ER was the first evidence that these two organelles could physically interact; these subcellular fractions have been named MAMs (mitochondria-associated membranes), and are the biochemical counterpart of MERCs [20, 21].

**Table 1:** Overview of the analyses performed in this study along with the cohorts of mice used. Timing shows the analysis relative to CSD (day)

Cohort no	Analysis	Timing (day)	CTRL (*n*)	CSD (*n*)	Σ (*n*)	Fig. panel
1	Blood glucose	48 h	10	12	22	1B
2	Glycogen	7 day	6	13	19	1C
3	MAMs	7 day	4	4	8	2/3A
4	HK1 and HK3	7 day	5	5	10	3B/4A, B/5A
5	HK3—cellular fractions	7 day	14	15	29	4C/5B

The idea that MERCs/MAMs are important for metabolic responses originates from several findings. First, the exchange of Ca^2+^ between ER and mitochondria is fundamental to maintain cellular bioenergetics under normal conditions [22]. Second, MFN2 ablation, and as a consequence the lowering of contact sites, severely impairs anorexigenic pro-opiomelanocortin (POMC) processing and increases ER stress, leading to the development of leptin resistance, decreased energy consumption, and obesity [23, 24]. The relevance of mitochondrial contact sites for the overall cell, organ, and finally whole organism metabolism also stems from the observation that in the liver from obese mice (either genetic models or obtained upon high-fat diet supplementation), MAMs increase in number, leading to oxidative stress and impaired glucose homeostasis [25]. This is further corroborated by the demonstration that contact sites among mitochondria and rough ER are important to maintain systemic lipid homeostasis in the liver [26].

Conversely, ablation of PACS2 or IP3R reduces oxidative stress and rescues glucose metabolism [25]. This evidence is in line with previous findings [27], showing that in postprandial conditions, the mouse liver is characterized by MERC expansion and, in parallel, with changes in mitochondrial cristae and decreased mitochondrial respiratory capacity (about 20% lower), in an MFN2-dependent manner. Liver-specific ablation of MFN2 has been shown to disrupt hepatic metabolism as well [28]. Therefore, MFN2 links mitochondrial and endoplasmic reticulum function with insulin signaling and is essential for normal glucose homeostasis. Overall, this evidence suggests that MERCs are highly plastic and rapidly alter their function to match changing metabolic needs.

In this work, we hypothesized that CSD stress results in cellular dysfunction by affecting the composition of MERCs and analyzed the proteome of MAMs from control and CSD mice using label-free quantitative proteomics. This revealed several differentially regulated proteins among which hexokinase 3 (HK3), a glucose-metabolizing enzyme, was studied in more detail. HK3 phosphorylates glucose to produce glucose-6-phosphate as the first step in glucose metabolism. As mitochondria tend to cluster in high concentrations in synaptosomes [29], we also investigated HK3 levels in this cellular compartment. Our work suggests that in healthy mice, HK3 is capable of translocating to MAMs and synapses in response to changes in blood glucose while this is abolished in CSD-stressed mice. This lack of intracellular glucose-induced trafficking to sites of need might contribute to a cellular dysfunction instigated by chronic stress.

## Materials and methods

### Animals

Male C57BL/6 J mice (*n* = 88, Janvier) arrived at 8 weeks of age in our animal facility (temperature = 22 ± 2 °C, relative humidity = 50 ± 5%). Upon arrival, mice were single-housed with food/water ad libitum in a light–dark cycle with lights on between 07:00 and 19:00. Animals were habituated to their new housing conditions for 1 week prior to experiments and belonged to one of five cohorts (Table 1). Male CD-1 mice (Janvier) at least 12 weeks of age upon arrival were retired breeders and used as aggressors in the CSD paradigm. Before experiments commenced, the CD-1 mice were tested for sufficient aggressive behavior.

### Chronic social defeat

The social defeat paradigm is well established [14, 30]. All animal studies were conducted in accordance with national guidelines and approved by the appropriate animal protection committees (Landesuntersuchungsamt RLP, 23 177 07/G 20–17-058). Briefly, C57 mice were either exposed to CSD or kept as controls. CSD consisted of a social defeat in the home cage of the aggressor mouse (CD-1) lasting 10 s of aggressive encounter. These episodes were repeated thrice, with different aggressors, interspersed by intervals in which the intruder and aggressor were separated through a perforated metal grid, which removed the somatosensory component of the social interaction. Following the daily triple social defeat, C57 mice were overnight housed with their opponent separated by the metal grid. The CSD paradigm lasted for 10 consecutive days. Control mice were daily placed in a novel cage for 90 s for the same 10-day period as CSD-treated mice. Similarly, during this period, control mice were housed in pairs in which the individuals were separated by a metal grid, thus closely mimicking the conditions experienced by the defeated mice. After CSD (or the control conditions), mice were again single-housed in a novel cage. No animals required exclusion for excessive wounding. The week following CSD, animals were sacrificed by decapitation after which the brain was rapidly removed.

### Peripheral blood glucose measurement

Mice were fasted for 1 h to exclude an immediate postprandial effect rise of blood glucose. Then, peripheral blood was obtained from the tail vein by a small nick using a scalpel under non-restrained conditions as before [31]. The first blood drop was discarded, and morning blood glucose levels were taken thrice (AccuChek®, Roche, Switzerland) and averaged to obtain reliable values.

### ELISA for hepatic glycogen measurement

Liver tissue was dissected and stored at − 80 °C until processing. Tissue was homogenized in 200 µL ddH2O and boiled for 10 min to inactivate the enzymes. The supernatant was processed according to the manufacturer’s instructions. Glycogen levels were measured in liver tissue using the Glycogen Assay Kit II (colorimetric, ab 169,558, Abcam) at 450 nm with a microplate reader (MultiScan FC, Thermo Scientific).

### Brain fractionation

Whole brains were extracted and cut in halves. One-half of the brains were used for MAM isolation, and the hippocampus, cerebellum, cortex, and striatum were dissected from the remaining brain halves. For MAM isolation, half brains were homogenized in isolation buffer (IBB; 225 mM D-mannitol, 75 mM sucrose, 30 mM Tris/HCl pH 7.4, 1 mM EGTA, 10 mM HEPES) and Percoll medium (225 mM D-mannitol, 25 mM HEPES/KOH pH 7.4, 1 mM EGTA, 30% (v/v) Percoll) were prepared. Reagents were obtained from Sigma-Aldrich. The fractionation of murine brain tissues was performed using an adjusted and optimized protocol, based on previously described methods [32, 33]. Before starting the experiment, buffers and the homogenizer with the teflon pestle were pre-cooled. Whole brains were transferred into 50-mL screw cap centrifugation tubes, washed with PBS 1 ×  + EDTA 10 mM and then cut into small pieces and trypsinized for 30 min at 4 °C. After a centrifugation step at 700 g (4 °C, 10 min), the brain pieces were resuspended into ice-cold IBB and put into a glass Teflon homogenizer. After homogenization (10 strokes at 1500 rpm on ice), samples were centrifuged at 800 g for 5 min at 4 °C and the supernatant fraction was collected. Lysate fractions were obtained after one additional centrifugation at 800 g for 5 min at 4 °C. The pellet was instead resuspended in IBB PNS fraction. A further centrifugation step led to separation of the mitochondrial pellet from the cytosolic fraction (from which microsomes and ER fractions were obtained upon centrifugation at 20,000 g for 30 min at 4 °C). Mitochondria were then gently resuspended in IBB, centrifuged twice at 8000 g for 10 min at 4 °C (crude mitochondria). The pure mitochondria fraction was then obtained upon ultracentrifugation through a Percoll gradient (swing-out rotor at 95,000 g for 30 min at 4 °C, “slow brake” mode, i.e., 10 min of deceleration). Following this step, the MAM fraction was visible as a dense white band in the middle of the gradient, and the pure mitochondria fraction being visible as a yellow band close to the bottom of the tube.

The hippocampus, cerebellum, cortex, and striatum were suspended in RIPA buffer and homogenized with a glass pestle. The samples were placed in a shaker and agitated for 2 h at 4 °C. After that, the samples were centrifuged at 20,000 g for 20 min at 4 °C. The supernatants were recovered, and the protein concentration was determined with the BCA assay.

### Isolation of crude synaptosomes from brain tissues

Crude synaptosomes were isolated using the Syn-PER Synaptic Protein Extraction Reagent (Thermo Scientific, 8779) according to the manufacturer’s instructions. Briefly, striatal tissues (left and right for each animal) were homogenized in 400 µL Syn-PER reagent containing protease (Protease Inhibitor Cocktail Complete™, Roche, Germany) and phosphatase inhibitors (Phosphatase Inhibitor Cocktails PhosSTOP, Roche, Germany) using a small pellet pestle (Motor Cordless Kimble). The homogenate was centrifuged at 4 °C and 1200 g for 10 min. The supernatant S1 was collected and centrifuged at 15,000 g for 20 min at 4 °C. Supernatant S2, containing the cytosolic fraction, was collected and pellet P2 (enriched in crude synaptosomes) resuspended in 70 µL of Syn-PER reagent containing protease and phosphatase inhibitors. Protein concentrations were measured using the BCA protein assay kit (Thermo Scientific, 23,225).

### Immunoblotting

The protein amount of the samples was quantified via the BC assay (Interchim). Denatured lysates (95 °C, 5 min) were then separated under reducing conditions on 4–15% Mini-PROTEAN® TGX Stain-Free™ gels (Bio-Rad) and subsequently transferred onto nitrocellulose membranes using the Trans-Blot® Turbo™ transfer system (Bio-Rad). Blocking of the membranes was conducted with 3% (w/v) milk powder in TBS-T (1 × TBS, 0.05% (v/v) Tween 20) for 1 h at room temperature (RT). The Chameleon Duo pre-stained protein ladder (LI-COR) served as protein size standard. The membranes were probed with the following primary antibodies: VDAC1 (voltage-dependent anion channel 1, Abcam ab14734), MFN2 (mitofusin 2, Abnova H00009927-M03), calnexin (Abcam ab22595), GRP75/mortalin (Neuromab 75–127), COXI (cytochrome c oxidase subunit 1, Abcam 14,715), COXIV (cytochrome c oxidase subunit IV, Cell Signaling 4844), hexokinase 3 (antibodies online, ABIN392753), hexokinase 1 (Cell Signaling C35C4), GAPDH (Abcam, 1:5000), synaptophysin 1 (synaptic systems, 1:1000, O/N), PSD95 (Cell Signaling Technology, 1:1000, O/N), and beta-III tubulin (R&D Systems, MAB1195 or Sigma-Aldrich, 1:500 for membrane fractions and 1:5000 for cytosolic fractions). Anti-mouse or anti-rabbit IgG (H + L) (DyLight 680 and 800 Conjugate, New England Biolabs) served as secondary antibodies and were diluted 1:15,000 in 3% (w/v) milk powder in TBS-T. Membranes were detected with the Odyssey infrared imaging system (LI-COR).

### Protein digest and LC–MS analysis

Samples were prepared for LC–MS using the previously described SP3 protocol [34]. For LC–MS analyses, a NanoAQUITY UPLC system (Waters Corporation, Milford, MA) was connected online to a Synapt G2-S high-definition mass spectrometer (Waters Corporation) through a NanoLockSpray dual electrospray ion source (Waters Corporation). Samples of 200 ng, which were tryptically digested, were loaded onto a HSS-T3 C18 1.8 μm, 75 μm × 250 mm reversed-phase analytical column (Waters Corporation) heated at 55 °C. Mobile phase A consisted of 0.1% (v/v) FA and 3% (v/v) DMSO in water and mobile phase B of 0.1% (v/v) FA and 3% (v/v) DMSO in ACN. To separate peptides, a 90-min running gradient from 5 to 40% (v/v) mobile phase B was used at a flow rate of 300 nL/min. Eluting peptides were analyzed by MS using ion mobility separation (IMS) as described before [35]. In short, precursor ion information was gathered in low-energy MS mode at constant collision energy of 4 eV, while fragment ion information was collected in the elevated energy scan applying drift-time specific collision energies. With 0.6 s for the spectral acquisition time in each mode and 0.05-s interscan delay, the overall cycle time was 1.3 s for the acquisition of one cycle of low and elevated energy data. As lock mass [Glu1]-fibrinopeptide was used at 250 fmol/µL, with a flow rate of 1.5 µL/min and analyzed every 30 s. The lock mass entered the MS via the reference sprayer of the NanoLockSpray source.

### Data processing and label-free quantification

The symphony (Waters ver. 1.0.0.191) pipeline was used for raw data processing and database search of LC–MS data. The database was custom made from murine proteome from UniProt (UniProtKB release September 2018, 16,991 entries), common contaminants, and reverse database entries. Search parameters included at least 2 fragment ions that had to be detected for peptide identification. For a protein to be reported, a minimum of 5 fragments summed over all assigned peptides had to be monitored. Furthermore, missed cleavages were set to 2, protease to trypsin, fixed modifiers to carbamidomethylation at cysteine, and variable modifiers to oxidation at methionine. The target decoy strategy was applied to determine the false discovery rate (FDR) for peptide and protein identification and was set to 0.01. The data of the experimental replicates of each condition were post-processed using the software ISOQuant ver. 1.8 including retention time alignments, exact mass retention time (EMRT) and IMS clustering, normalization, and protein homology filtering. Details have been described [35]. Additionally, the following settings were used: minimum peptide length of 6 amino acids, minimum 2 peptides per protein, minimum max score per cluster 6, FDR 0.01, no missed cleavage, and no variable modifications, e.g., methionine oxidation. Of those proteins with significant abundance difference, the proteins needed to have at least 3 consecutive amino acids in the peptide fragments identified, max score > 1000, and be reported in at least 2 out of the 4 measurements per condition. Absolute sample amounts were calculated for each protein using TOP3.

### Statistics

All samples represent biological replicates. The experiments and analyses were performed in a blinded fashion. Sample sizes are indicated in the figure legends. Data are expressed as mean ± SEM. A *p* < 0.05 was considered statistically significant. Data were checked for normal distribution using the Kolmogorov–Smirnov test. Unpaired two-tailed Student’s *t* test (normally distributed) or Mann–Whitney *U* test (not normally distributed) were used to compare sets of data obtained from two independent groups of animals. Pearson’s or Spearman’s (non-parametric) correlation coefficient was used to measure linear correlation between two sets of data. Except for proteomics data (previous section), all data were analyzed using Prism (GraphPad Software).

## Results

### Chronic social defeat results in profound changes of glucose metabolism

**Fig. 1:**
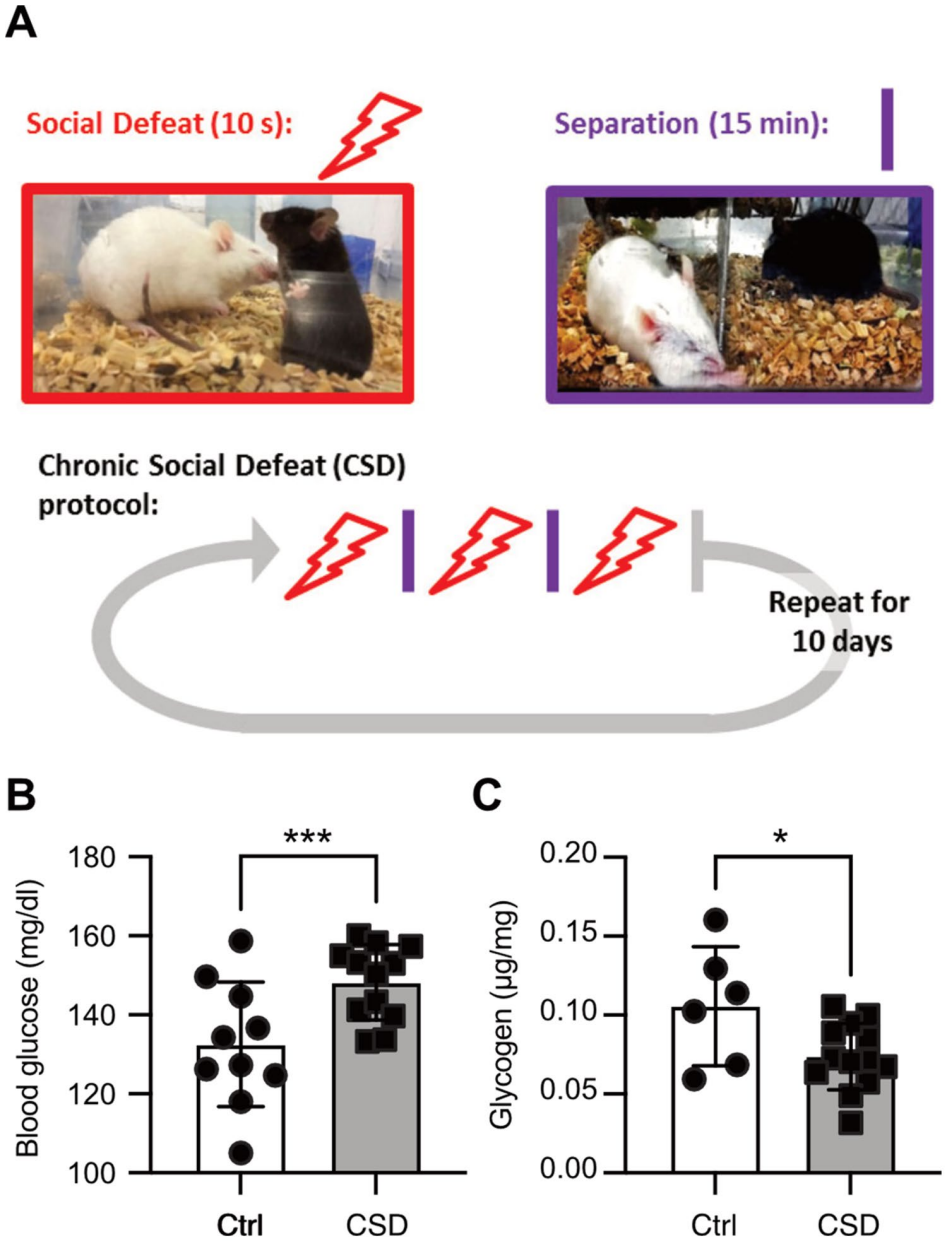
Chronic social defeat results in profound changes of glucose metabolism. **A** The stressor consisted of a social defeat protocol lasting 10 consecutive days. Each day, stressed mice were subjected to three episodes of aggressive behavior lasting 10 s. In between aggressive encounters and overnight, animals were separated via a metal barrier, preventing further physical contact. Nevertheless, exposure to several other stressful aspects (smell, vision, and sound of the aggressor) lasted throughout the entire paradigm. **B** Peripheral blood glucose levels were increased in stressed mice 48 h post-CSD (*p* = 0.0094) while **C** hepatic glycogen concentrations were reduced 1 week post-CSD (*p* = 0.028). The mice described in **B** and **C** correspond to different cohorts not studied simultaneously (mice in **B** to cohort #1 and mice in **C** to cohort #2). Bars show the mean ± SEM with each data point representing one mouse. *p < 0.05; **p < 0.01; ***p < 0.005; Student’s *t* test

Mice were exposed to CSD (Fig. 1A) or treated as controls. Control mice were also placed in new cages and pair-housed separated by a metal grid, thus closely mimicking the conditions experienced by the defeated mice. CSD results in profound changes of glucose metabolism as previously reported [14]. Here, we confirmed that peripheral blood glucose levels are significantly upregulated upon CSD (Fig. 1B). Additionally, we observed that hepatic glycogen levels were diminished 1 week post-CSD (Fig. 1C). Hence, the peripheral hyperglycemia seen in CSD-exposed mice may be a consequence of increased hepatic glycogenolysis.

### Characterization of subcellular fractions from whole brains obtained from mice exposed to chronic social defeat and control animals

**Fig. 2:**
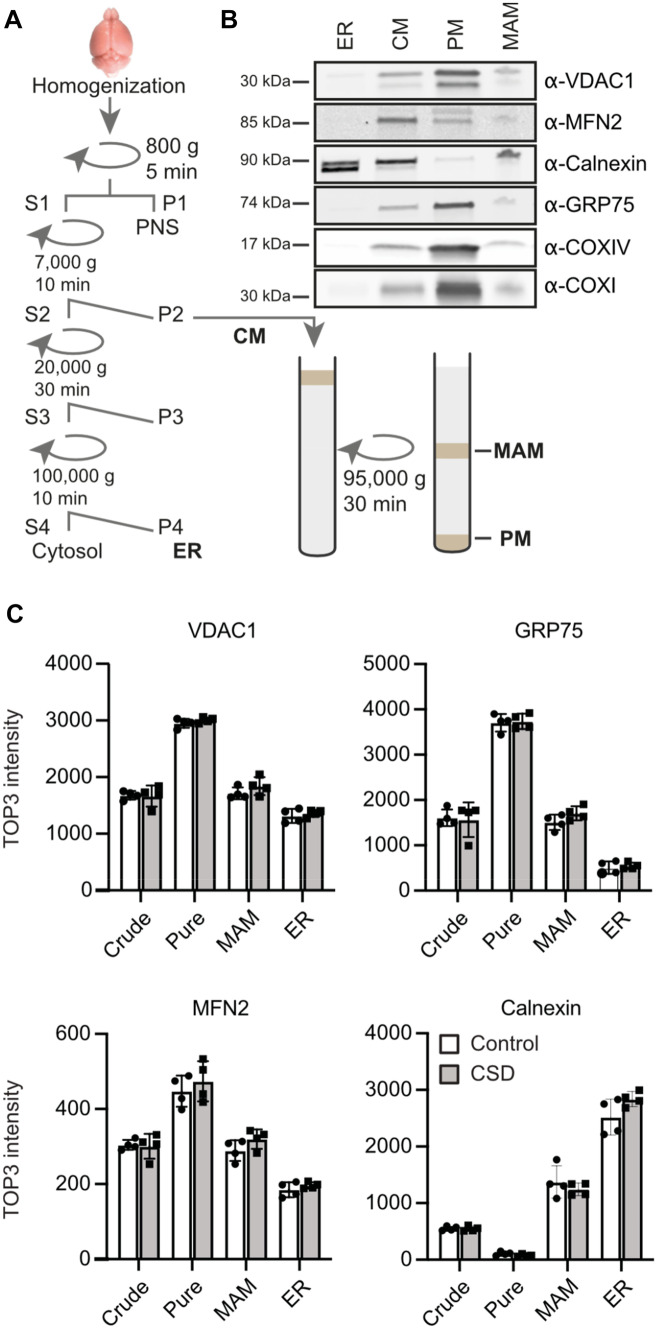
Fractionation procedure and verification. **A** Schematic overview of MAM and mitochondrial enrichment by differential centrifugation after homogenizing whole brains from control of CSD-exposed mice. ER, endoplasmic reticulum; CM, crude mitochondria; PM, pure mitochondria; MAM, mitochondria-associated membranes; S, supernatant; P, pellet. **B** Verification of enriched cellular fractions by immunoblot against VDAC1, MFN2, calnexin, GRP75/mortalin, COXI (cytochrome c oxidase subunit 1), and COXIV (cytochrome c oxidase subunit IV). **C** Validation subcellular fractioning: as expected, the mitochondrial MAM markers VDAC1, GRP75 and MFN2 levels are most abundent in the pure mitochondrial fraction but were also increased in the MAM fraction (in comparison to ER). In contrast, calnexin, a MAM marker from the ER, was most abundant in the ER fraction, but was also increased in the MAM fraction (as compared to the pure mitochondrial fraction). Bars show the mean ± SEM with each data point representing one mouse

Seven days post-CSD, we removed and processed the brains from CSD and control mice (cohort #3). Fractions containing the ER, the MAMs as well as pure and crude mitochondria were obtained by differential centrifugation and sucrose gradients (Fig. 2A), and analyzed by immunoblotting to verify their purity. We used antibodies against cytochrome c oxidase I (COXI) and IV (COXIV) as markers for mitochondria. GRP75/mortalin, VDAC1, and MFN2 are well-known MAM markers mainly localized at the outer mitochondrial membrane, and calnexin is a MAM marker from the ER (Fig. 2B).

These MAM proteins were also quantified using label-free proteomics. As expected, VDAC1, MFN2, and GRP75/mortalin were more abundant in the pure mitochondria fraction while calnexin was most abundant in the ER fraction (Fig. 2C). Importantly, none of these proteins were dysregulated in CSD mice. We conclude that our subcellular fractionation worked and that CSD does not affect the abundance of MAM marker proteins in these fractions.

### Hexokinase 3 is absent in the mitochondrial-associated membrane fraction from CSD mice

**Fig. 3:**
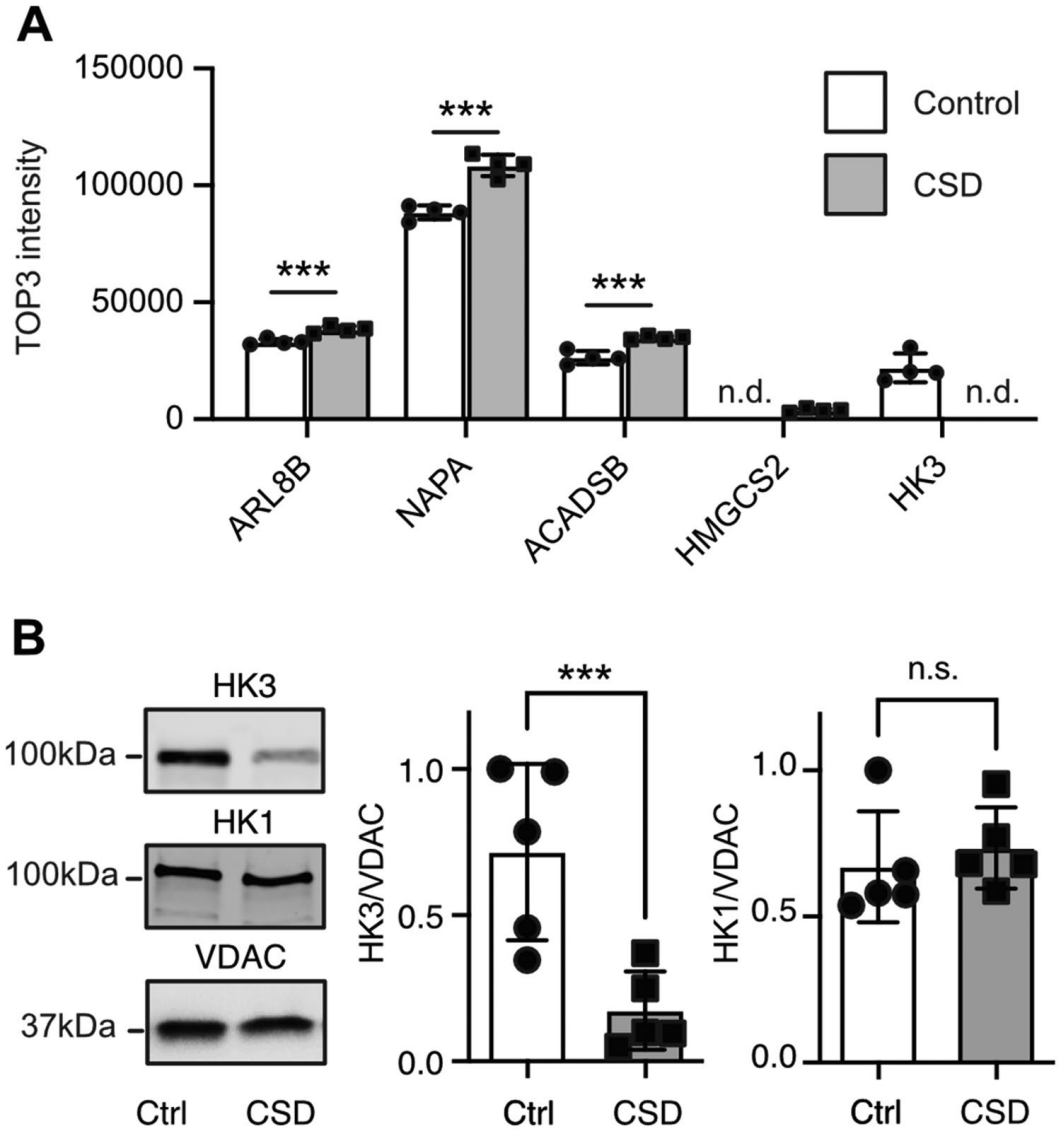
Hexokinase 3 is downregulated in MAMs from CSD mice. **A** The bar graphs show the mean ± standard deviation TOP3 intensity with all individual data points obtained by label-free proteomics from subcellular fractions containing mitochondria-associated membranes from cohort #3 mouse brains. All differences are statistically significant using the two-stage linear step-up procedure of Benjamini, Krieger, and Yekutieli with a false discovery rate (*Q*) of 1% to control for multiple testing. n.d., not detected. **B** Reproduction of the findings with a different cohort (#4) of mice. Hexokinase 1 (HK1) served as control. Exemplary immunoblots and quantification. Size is indicated. Bars show the mean ± SEM with each data point representing one mouse. *p < 0.05; **p < 0.01; ***p < 0.005; n.s., not significant; Student’s *t* test

We next sought to identify regulated proteins within the MAM fractions. All proteins shown and discussed below were reliably detected with a score > 1000 in all four samples. Those identified by conventional *t* testing were further subjected to a second analysis using the two-stage linear step-up procedure of Benjamini, Krieger, and Yekutieli with a false discovery rate (*Q*) of 1% to control for multiple testing [36]. This stringent procedure resulted in only five proteins that were differentially regulated in mice exposed to CSD as compared to controls. Three were significantly upregulated in the CSD brains, while one protein was only detected in MAMs from CSD brains, and one only detected in MAMs from control brains (Fig. 3A).

Proteins upregulated in CSD MAM fractions were the lysosomal protein ARL8B (ADP ribosylation factor like GTPase 8B) [37], increased 1.16-fold (*p* = 0.0017), and NAPA (*N*-ethylmaleimide-sensitive factor attachment protein alpha) (1.23-fold, *p* = 0.0003), a member of the SNAP (soluble NSF attachment protein) family which plays a critical role in the docking and fusion of vesicles to target membranes [38, 39]. Also, the protein ACADSB (acyl-CoA dehydrogenase short/branched chain) was upregulated 1.32-fold up in CSD MAMs, *p* = 0.0012) while HMGCS2 (3-hydroxy-3-methylglutaryl-CoA synthase 2) was only found in MAMs from CSD mice. We decided to focus further on hexokinase 3 (HK3) because it was the most regulated protein and because of its direct involvement in glucose metabolism. Like all hexokinases, HK3 phosphorylates glucose to produce glucose-6-phosphate (G6P) as the first step in glucose metabolism. HK3 was not present in MAM fractions from CSD mice. We reproduced this downregulation of HK3 in an independent cohort (#4) of animals. HK1, in contrast, which we used as control, was not regulated (Fig. 3B).

### The reduction of HK3 in MAMs in CSD mice is not caused by an overall downregulation or a shift to synaptic sites

**Fig. 4:**
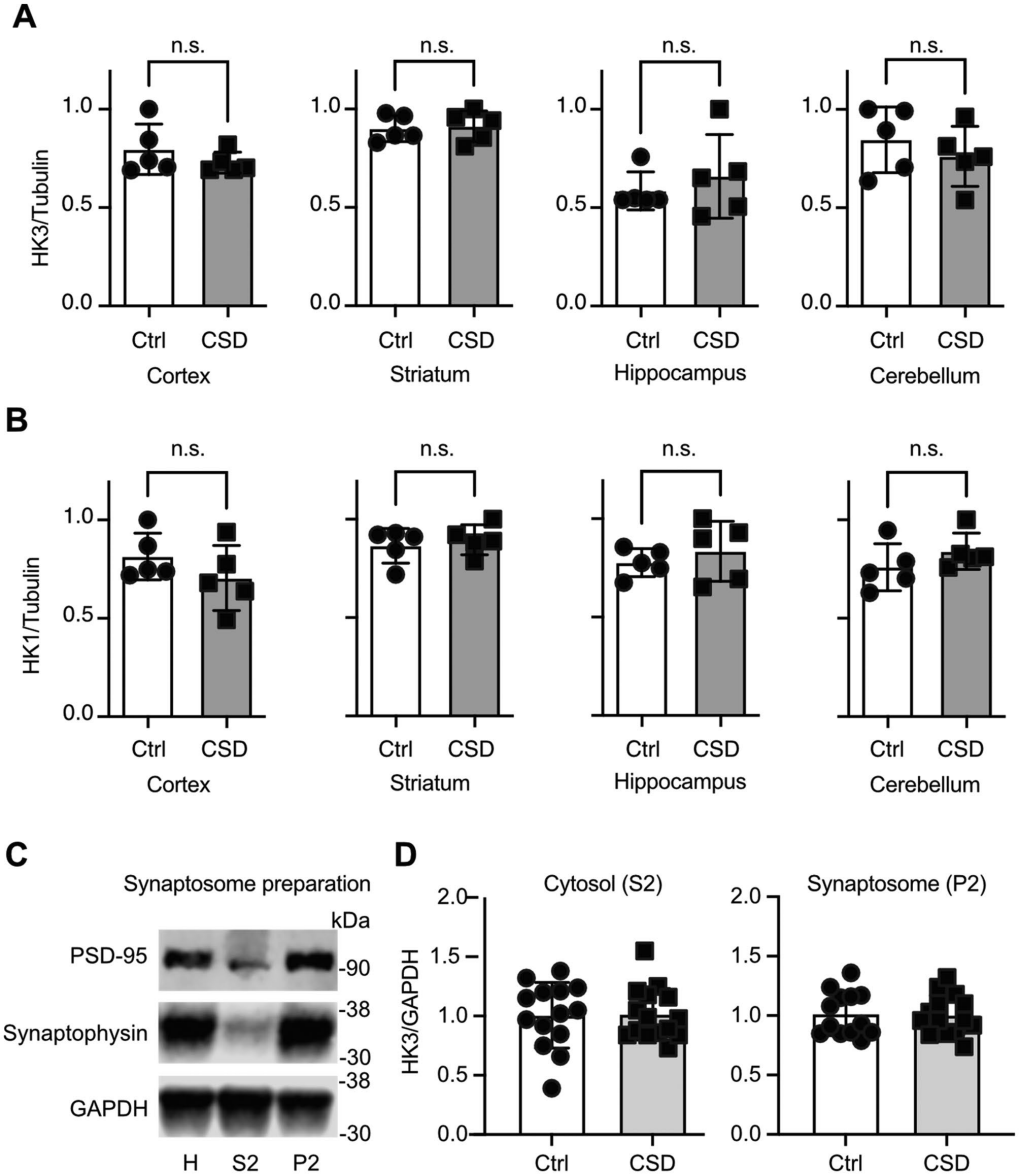
The reduction of HK3 in MAMs in CSD mice is not caused by an overall downregulation or a shift towards synaptic sites. Immunoblots using tissue from the indicated brain regions from cohort #4 mice stained with antibodies against **A** HK3 and **B** HK1. Size is indicated. β-Tubulin III served as loading control. **C** Synaptosomes obtained by differential fractionation. **D** Immunoblot quantification from cohort #5 mouse brains. Bars show the mean ± SEM with each data point representing one mouse. Student’s *t* test, n.s., non-significant

To rule out that HK3 is simply downregulated in CSD mice and to clarify where in the brain it is most abundant, we immunoblotted brain tissue from cortex, striatum, hippocampus, and cerebellum from CSD and control mice with antibodies against HK3. HK1 served again as control. These tissues were derived from the other hemisphere of the same cohort used for the MAM analysis (#4). The immunoblots demonstrated that both proteins are similarly abundant in control and CSD mouse brains (Fig. 4A). It is technically very difficult to obtain MAM fractions from these parts of the brain; we could therefore not clarify whether the HK3 downregulation in MAMs is specific to one or several brain regions. It is, however, possible to isolate synaptosomal fractions. We therefore decided to study whether HK3 translocates to synaptic sites in CSD mice, which could also explain the apparent reduction in MAM fractions because synapses contain mitochondria but not a lot of ER.

For this, we used striatal tissue because HK1 and HK3 displayed the strongest expression in the striatum (Fig. 4A, B), a tissue with strong energy demands due to its abundance of input, glutamatergic input from both the cortex and thalamus as well as dopaminergic input from the midbrain. Synaptosomal fractions were generated from another cohort of mice (#5) and characterized using immunoblotting against the synaptic protein markers synaptophysin and PSD95 (Fig. 4C). This revealed that striatal synaptosomes from control and CSD mice contained similar amounts of HK3 (Fig. 4D). We concluded that HK3 is less abundant specifically in MAM fractions from CSD mice and that this result is not caused by a general downregulation or translocation of HK3 to synaptic sites.

### The ability of hexokinase 3 to associate with mitochondria-associated membranes and synapses in response to increases in blood sugar is lost in CSD mice

We next asked whether the absence of HK3 from MAMs in CSD mice correlates with blood glucose levels. As mentioned above, blood glucose levels are elevated in CSD mice, but this is not matched by increased glucose tissue uptake, as shown in previous work using positron emission tomography [14]. This finding already suggested that brain tissue somehow restricts glucose uptake, possibly to avoid excessive and potentially detrimental levels.

**Fig. 5:**
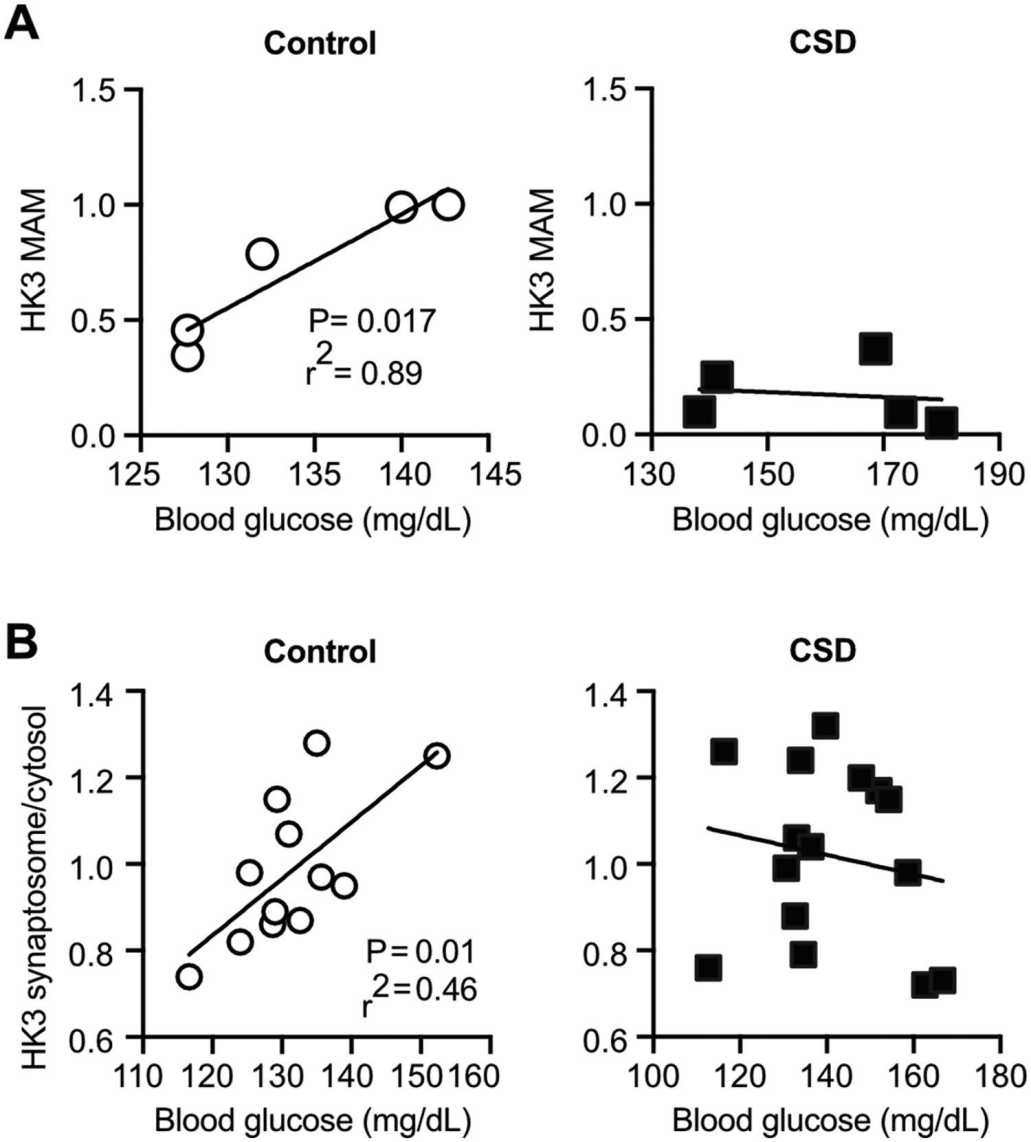
No correlation between hexokinase 3 and peripheral glucose concentrations in CSD animals. **A** Peripheral blood glucose levels, taken 48 h post-CSD, correlated positively with the amount of HK3 protein in the MAMs in controls, but not in stressed mice from cohort #4. **B** Peripheral blood glucose levels correlated positively with the ratio of synaptosomal versus cytosolic HK3 in controls but not in stressed mice from cohort #5. Each data point represents one mouse. Statistical significance was calculated using the Pearson’s coefficient

When we plotted the abundance of HK3 in MAMs from cohort #4 (Fig. 5A) and in synaptosomes from cohort #5 (Fig. 5B) against the individual blood glucose levels, we noted a significant positive correlation between these two parameters in control mice. HK3 therefore translocates to MAMs and synapses—potential sites of need—in response to higher substrate levels. This correlation is, however, lost in CSD mice.

## Discussion

We here describe that chronic social defeat (CSD) stress affects the composition of mitochondria-associated membranes (MAMs), the biochemical counterpart of mitochondria-ER contact sites in the brain. Herein, hexokinase 3 (HK3) emerged as an important metabolic regulator. In control mice, HK3 in MAMs and in mitochondria-rich synaptosomes were closely matching the peripheral blood glucose levels. These correlations are lost in CSD mice. In fact, HK3 was severely diminished in MAMs from CSD-exposed mice. One possible interpretation is that intracellular HK3 trafficking in the brain is important in trapping glucose and supplying mitochondria with an adequate amount of glucose-6-phosphate (G6P) for further metabolism and that this process is disrupted upon CSD. G6P can be used in glycolysis and produce pyruvate as fuel for the TCA. Alternatively, G6P can also be diverted into the pentose phosphate pathway (PPP), where it serves to generate the reducing agent NADPH, which is required for the production of reduced GSH, a major scavenger of reactive oxygen species [40]. Since chronic stress shifts the redox balance to a more oxidative state [41–43], increased reducing capacity generated by G6P fueling of the PPP could serve as a compensatory mechanism.

Previously we reported that CSD results in peripheral hyperglycemia and that the stressed brain is hyperglycemic [14]. In the current study, we replicated the CSD-induced peripheral hyperglycemia, which were at levels comparable to those reported previously [14, 30]. Although we now only measured peripheral glucose levels, our earlier work demonstrated that peripheral and central glucose concentrations correlate positively [14]. We are therefore convinced that the elevated glucose concentrations in the peripheral blood of CSD-exposed mice reported here can be extrapolated to the brain. In our earlier work, we also observed that, despite increased blood glucose levels, intracellular glucose uptake in the brain is compromised in CSD-exposed mice [14]. Hence, we assume that stress-induced cerebral hyperglycemia does not represent an adaptive response meeting increased glucose demands. Rather, the stressed brain is vulnerable to excessive glucose [6, 44] and likely aims to limit its intracellular flux, for instance by reducing glucose transport, as reported previously for GluT1 [14]. In this vein, we hypothesize that CSD restricts intracellular HK3 trafficking towards mitochondria in an attempt to block excessive glucose metabolism. The downregulation of HK3 was specific to the MAM fraction; whole homogenates from cortex, striatum, hippocampus, and cerebellum did not display differences in HK3 abundance between controls and CSD. Moreover, the disrupting effects of CSD appear to be selective for the HK3 MAM connection since HK1 MAM levels were unaffected. Interestingly, HK3 does not contain a mitochondrial targeting signal like HK1 and HK2 [45], but is still found in the MAM fraction of the brain. While HK3 is a rather less well-studied member of the hexokinase family, other hexokinases directly interact with VDACs [46], a key constituent of MAMs, thereby regulating aerobic glycolysis (reviewed in [47]). Ectopic overexpression of HK3 in vitro was shown to be protective against oxidative stress and preserved mitochondrial integrity [45], in line with the protective properties reported earlier for HK1 and HK2 [48]. It remains to be determined to which extent HK1- and HK3-mediated actions have divergent consequences for glucose metabolism. Although the mechanisms underlying intracellular HK3 translocation, and their stress-induced perturbations, are unclear, we surmise that MAMs and synapses constitute sites of need for increased glucose metabolism when abundant substrate is present. Accordingly, we imagine that HK3 in non-stressed individuals could play a role in glucose sensing [49].

MERCs constitute important intracellular signaling hubs and nutrient sensors and allow the efficient exchange of lipids and calcium between the two organelles. MERCs are indispensable for the correct functioning of mitochondria, the main energy source of eukaryotic cells. Interestingly, three out of five dysregulated MAM proteins upon CSD are involved in metabolism. Two of these proteins were increased in MAMs from stressed animals namely ACADSB, a member of the acyl-CoA dehydrogenase family of enzymes [38], and HMGCS2, a HMG-CoA synthase protein that catalyzes the first reaction of ketogenesis, a metabolic pathway that provides lipid-derived energy for various organs during times of carbohydrate deprivation like fasting [50]. Interestingly, both upregulated proteins are involved in alternative pathways of acetyl coenzyme A (acetyl-CoA) production besides glycolysis where glucose is broken down to two molecules of pyruvate and further catalyzed by the pyruvate dehydrogenase complex (PDC) to acetyl-CoA. In the alternative pathway, acetyl-CoA is generated from fatty acids through β-oxidation. The first step of β-oxidation is the dehydrogenation of acyl-CoA by dehydrogenases like ACADSB. After several other steps and transport into the mitochondrial matrix, the end product of β-oxidation acetyl-CoA is fed into the tricarboxylic acid cycle (TCA) to run the mitochondrial electron transport chain and ultimately produce energy. However, if the amount of acetyl-CoA generated is not met by the processing capacity of the TCA cycle, acetyl-CoA can be used for the biosynthesis of ketone bodies. This reaction is catalyzed by HMGCS2, the other stress-upregulated protein. Taken together, the stress-induced upregulated proteins ACADSB and HMGCS2 are known to be activated by caloric restriction and suggest that the energy production in CSD-exposed animals may shift from oxidative phosphorylation to fatty acid degradation. Thus, we argue that, despite an abundance of nutrients in the form of glucose, the stressed brain may actually be starving.

To conclude, our results suggest that intracellular glucose metabolism is compromised in the stressed brain, despite or because of excessive blood glucose concentrations. While such adaptations probably represent defense mechanisms that protect the brain against a stress-induced glucose overload, they may simultaneously be harmful, resulting in neuronal dysfunction.

## Data Availability

The mass spectrometry proteomics data have been deposited to the ProteomeXchange Consortium (http://proteomecentral.proteomexchange.org) via PRIDE partner repository with the data set identifier PXD015983.
